# Magnetic anisotropy of graphene quantum dots decorated with a ruthenium adatom

**DOI:** 10.3762/bjnano.4.51

**Published:** 2013-07-10

**Authors:** Igor Beljakov, Velimir Meded, Franz Symalla, Karin Fink, Sam Shallcross, Wolfgang Wenzel

**Affiliations:** 1 Institute of Nanotechnology (INT), KIT, Karlsruhe, Germany; 2 Friedrich-Alexander-Universität Erlangen-Nürnberg (FAU), Erlangen, Germany

**Keywords:** adsorbate, grapheme, graphene quantum dot, magnetic anisotropy, transition metal

## Abstract

The creation of magnetic storage devices by decoration of a graphene sheet by magnetic transition-metal adatoms, utilizing the high in-plane versus out-of-plane magnetic anisotropy energy (MAE), has recently been proposed. This concept is extended in our density-functional-based modeling study by incorporating the influence of the graphene edge on the MAE. We consider triangular graphene flakes with both armchair and zigzag edges in which a single ruthenium adatom is placed at symmetrically inequivalent positions. Depending on the edge-type, the graphene edge was found to influence the MAE in opposite ways: for the armchair flake the MAE increases close to the edge, while the opposite is true for the zigzag edge. Additionally, in-plane pinning of the magnetization direction perpendicular to the edge itself is observed for the first time.

## Introduction

Since 2004, graphene [1], a one-atom-thick sheet of carbon atoms arranged in a regular hexagonal lattice, has been investigated intensively [2]. Outstanding mechanical and electronic properties, both predicted and measured [3], make it one of the most studied materials both theoretically and experimentally [4–11]. High magnetic anisotropies were predicted for graphene decorated with transition-metal (TM) adatoms and dimers [12–13]. Inspired by its application potential in the fields of spintronics and magnetic storage as well as fundamental science, a number of works were published on the properties of such structures [14–17]. The studies mostly describe two extreme cases of substrates, i.e., single benzene molecules, less suitable for a realistic device, because of their size and problematic realization (benzene is an easily flammable toxic liquid), or infinite graphene with a certain periodic coverage of metal adatoms. A homogeneous distribution of adatoms on a graphene sheet may pose further experimental difficulties, due to the possibility of adatom clustering. The magnetic anisotropy energy (MAE) is known to be profoundly influenced by the symmetry of the environment. On the other hand, graphene flakes both provide a natural interpolation between the two limits, i.e., the infinite graphene sheet and the benzene ring, which have already been studied in the literature, as well as provide a possible template for the adsorption of magnetic adatoms, by preferential adsorption at the edges. Considering the higher spin-orbit coupling of 4d TMs compared to 3d TMs, as well as the fact that the first observation of 4d ferromagnetism was made for a ruthenium monolayer on a graphite substrate [18], Ru appeared as an attractive candidate for the adatom. All of these reasons motivate our present study of the MAE of Ru adatoms on a graphene flake.

## Methods

As the system of choice, triangular hydrogen-saturated graphene flakes (or graphene quantum dots) were investigated, comprising 90 and 97 carbon atoms for two different edge types, armchair and zigzag (AGQD and ZGQD), respectively. The triangular shape was chosen as the simplest geometry, providing the same edge type on all sides. On a chosen hollow site (above a carbon ring center) a ruthenium adatom was placed, and the distance was optimized by minimization of the total energy using density functional theory. The stability of the Ru adatom on the hollow site, surrounded by less preferable positions over the C–C bridge and atop a C-atom, was reported for infinite graphene [15]. The graphene flake is considered fixed, as it would be on a substrate.

Within our work we used density functional theory (DFT) [19] with the B-P86 generalized gradient approximation (GGA) functional and the hybrid functional B3-LYP as implemented in TURBOMOLE [20–21]. The (Grimme) empirical dispersion correction (DFT-D2) [22] was used for geometry optimizations. To calculate the magnetic anisotropy we used the two-component calculation [23] with dhf-TZVP-2c basis [24]. For the spin–orbit interaction the two-component effective core potential dhf-ecp-2c [25] was used.

To determine the magnetic anisotropy of the system at hand, the magnetization direction was varied, and the resulting total energies were compared. Three magnetization directions and corresponding energies were used: (1) the out-of-plane (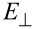) direction, pointing perpendicular to the flake plane; (2) the in-plane-minimum (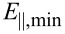) direction, i.e., the direction parallel to the flake plane with the lowest total energy; and (3) the in-plane-maximum (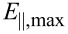) direction, for the highest total energy in-plane. Note, direction 2 may depend on the chosen site, and directions 2 and 3 are not necessary perpendicular to each other. Using the defined directions, the following two kinds of MAE are defined. The in- versus out-of-plane (*E*_IO_) MAE is defined as

[1]
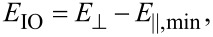


and is negative when the easy axis points out of plane. If *E*_IO_ is positive, the easy axis points along the direction 2. The in-plane MAE (*E*_IP_) is defined as

[2]
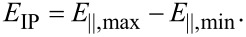


The *E*_IP_ is per definition always positive and would be equal to zero for an adatom on an infinite graphene sheet, due to the underlying symmetry.

## Results

**Figure 1:**
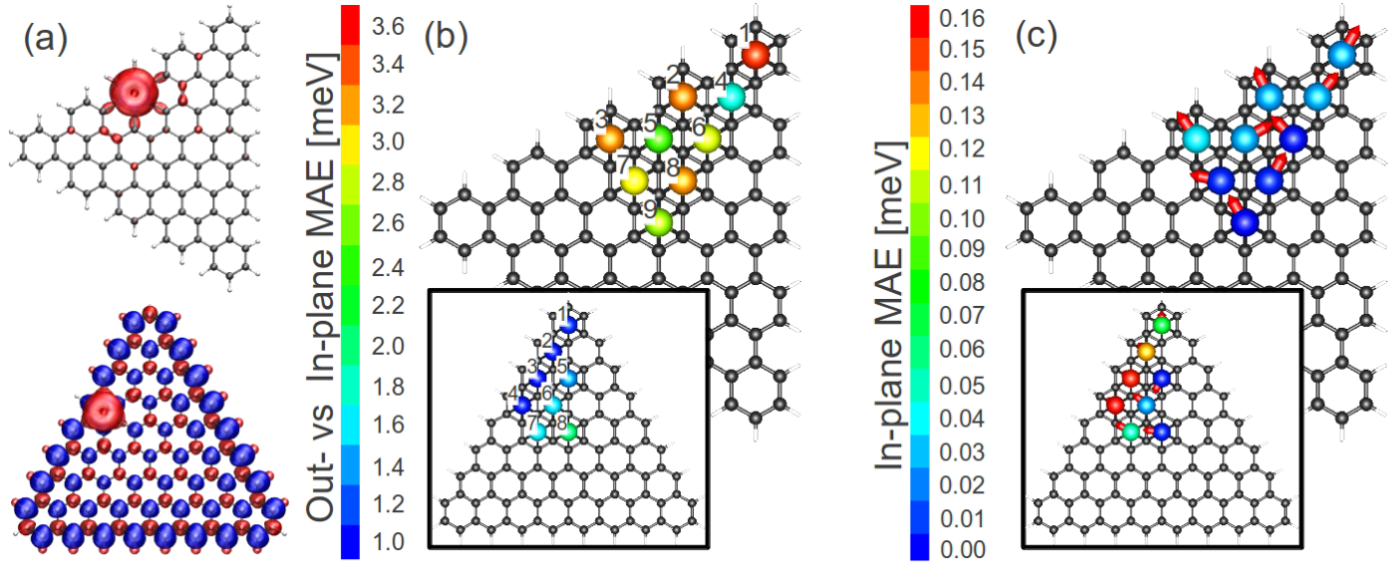
(a) The magnetization density of an armchair (top) and zigzag (bottom) graphene flake decorated with a single ruthenium adatom at a separation of 1.75 Å from the flake plane; positive (negative) *m*(r) indicated by the red (blue) colour. Shown in panel (b) are the in-plane versus out-of-plane magnetic anisotropy energies for all symmetry-inequivalent adatom positions, see Equation 1 for the definition of this quantity, for both the armchair and zigzag (as inset) graphene flakes. In panel (c) are similarly presented the in-plane magnetic anisotropy energies (see Equation 2), with the red arrows indicating the direction of the minimum-energy in-plane position.

We first consider the electronic structure of the armchair-graphene (AGQD) and zigzag-graphene (ZGQD) quantum dots in the pristine state, i.e., without decoration by a Ru atom. (The geometric structure may be seen in Figure 1.) While the AGQD has no intrinsic moment, the ZGQD in contrast is found to have an intrinsic moment of 7 μ_B_. This spin polarisation of the ZGQD arises from a highly localized *p*_z_-type edge state [26], and the total moment of the quantum dot is exactly equal to the difference in number between the A-type atoms and B-type atoms, in agreement with the theorem of magnetism in a bipartite lattice at half filling reported by Lieb [27]. As we shall subsequently see, this difference in the magnetic state of the pristine graphene quantum dots leads to a qualitatively different behaviour of the magnetic anisotropy of the absorbed Ru atom.

Before considering in detail the magnetic state of the Ru adatom, however, we shall consider the nature of its bonding to the graphene flake. We find the calculated Ru–flake separation to be 1.75 Å, strongly indicating chemisorption, a fact supported by the significant reduction in the Ru moment from the atomic state (we find the moment to be always less than 2 μ_B_ while the atomic moment of Ru is 4 μ_B_, see below), as well as the large binding energy of the Ru adatom which we find to be of the order of eV (see Table 1). In addition we note that the most energetically preferred position on the graphene flake is, for both the AGQD and ZQGD, the apex site (see Table 1). This finding is compatible with previous work on the absorption of transition-metal atoms on graphene nanoribbons [28].

**Table 1:** Total energy of the graphene flakes decorated with a Ru atom. The Ru atom was put on 9(8) sites with nonequivalent symmetry on armchair (zigzag) graphene flakes. The sites are numbered according to Figure 1b. As a zero-point system the corresponding flake was chosen with a Ru atom placed 100 Å away from the flake plane.

Position AGQD	*E*_Tot_ [eV]	Position ZGQD	*E*_Tot_ [eV]

1	−2.249	1	−2.369
2	−1.987	2	−2.036
3	−1.980	3	−2.026
4	−1.451	4	−2.031
5	−1.485	5	−1.697
6	−1.328	6	−1.637
7	−1.372	7	−1.628
8	−1.590	8	−1.568
9	−1.474		

We now turn to the question of the detailed magnetic structure of the graphene flake with the Ru adatom. Considering first the in-plane versus out-of-plane anisotropy (*E*_IO_) we find that (i) *E*_IO_ > 0 for all absorption positions on both flakes, i.e., the easy axis is in-plane and (ii) *E*_IO_ is significantly larger on the AGQD as compared to the ZGQD, see Figure 1b in which the *E*_IO_ is plotted for all 9 (8) symmetrically inequivalent sites of the AGQD (ZGQD).

**Figure 2:**
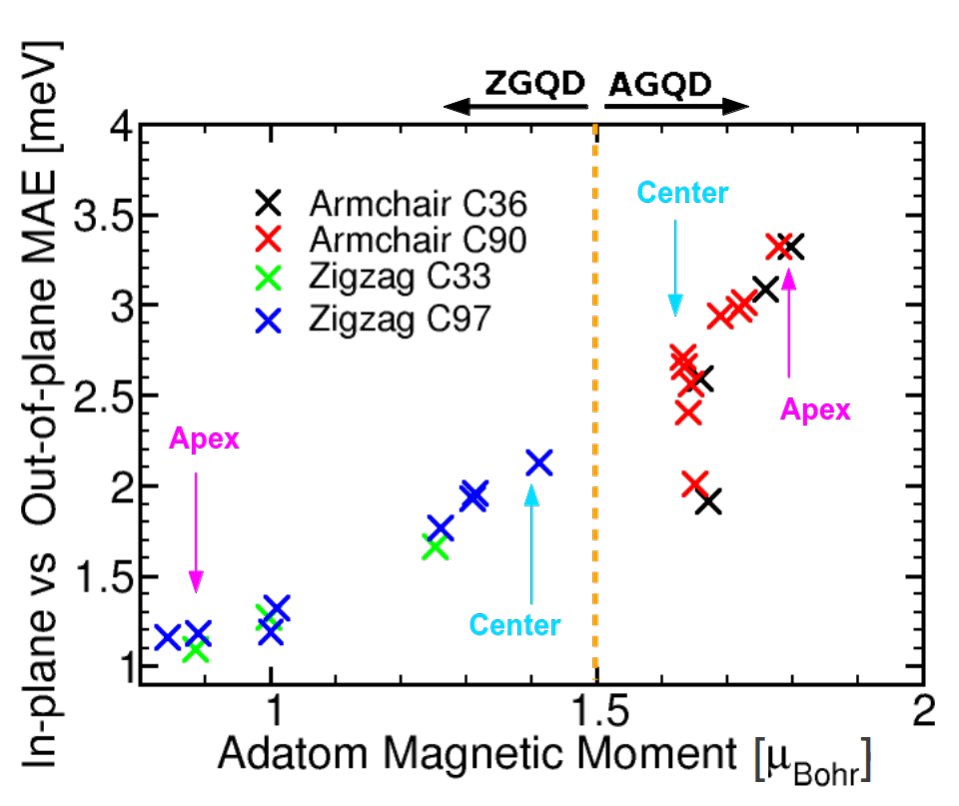
Correlation between the magnetic moment and the in-plane versus out-of-plane anisotropy, *E*_IO_, see Equation 1, for armchair-graphene quantum dots (AGQDs) and zigzag-graphene quantum dots (ZGQDs) consisting of 36 and 90 carbon atoms for the AGQDs, and 33 and 97 carbon atoms for the ZGQDs. Each of the points represents the spin moment and *E*_IO_ for an adsorbate position of the Ru adatom. Evidently, the larger the Ru moment the greater the value attained for *E*_IO_. Specific absorbate positions (edge, apex) are indicated by the text. Note that the edge positions of the ZGQD have the lowest adatom moment (and so lowest *E*_IO_) while, in contrast, on the AGQD these positions have the highest adatom moment and *E*_IO_. Points that deviate from the overall trend reflect a specific electronic structure associated with low symmetry positions of the AGQDs.

As we shall now demonstrate that the origin of this difference in the magnitude of *E*_IO_ between the two flakes can be traced back to the fact that, while the bare ZGQD is spin polarised, the AGQD is not. To this end we first note that spin coupling of the Ru adatom to the *p*_z_ spin-split edge state on the ZGQD is antiferromagnetic. This we illustrate in Figure 1a, in which isosurfaces of the magnetisation density (*m*(r)) for Ru on the AGQD and ZGQD are shown. Clearly while in the former case the Ru acts to weakly polarise the flake with ferromagentic coupling, in the latter case the coupling is strongly antiferromagnetic (note that blue and red indicate negative and positive *m* isosurfaces). The net result of this antiferromagnetic coupling is to strongly reduce the moment of the Ru adatom on the ZGQD: while moments of 1.7–1.8 μ_B_ are found for the AGQD, for the ZGQD these values fall to 0.8–1.4 μ_B_. To bring out the relation to the in-plane versus out-of-plane anistropy, *E*_IO_, we plot this quantity against the Ru adatom moment in Figure 2. As can be seen the clear trend that emerges is that the larger the moment the greater the value attained for the anisotropy. In this plot the impact of the AFM coupling of Ru is clearly made visible: while the Ru moment increases at the edge sites for the AGQD, as there are fewer C atoms with which to share the unpaired electrons of Ru, towards the edge sites of the ZGQD the moment, in contrast, is seen to decrease.

In short, the electronic structure of the graphene substrate determines the polarisation of the absorbed Ru atom and this in turn governs the value of *E*_IO_. This is, in fact, a rather natural result as the physics of anisotropy is, upon expansion of the Dirac equation in powers of *v*/*c*, governed by the spin–orbit coupling term which is proportional to 
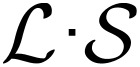
 with 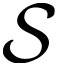 the spin moment.

Having thoroughly probed the physics of the in-plane versus out-of-plane anisotropy we now consider the question of which in-plane direction the Ru moment assumes, i.e., the question of what is the in-plane easy axis of the spin. To this end we calculated *E*_IP_ for all the symmetry-inequivalent absorbate positions, as shown in Figure 1c. As may be seen, *E*_IP_ is generally an order of magnitude smaller than *E*_IO_ and attains its maximum value, as one would expect, at the edges of the graphene flakes. This follows from the fact that it is the lowering of the symmetry of the local environment that is crucial for the anisotropy (as the existence of orbital currents implies local magnetism), and hence, in the centre of even the rather small flakes presented here the anisotropy is substantially lower than at the edges. Interestingly, we find that for the edge positions the spin always points perpendicular to the boundary of the flake.

## Conclusion

Using first-principles DFT methods we have investigated the magnetic properties of Ru adatoms on two types of graphene flakes: the armchair (AGQD) and zigzag (ZGQD) edged triangular graphene quantum dots. The geometry of these flakes is such that each has only one specific type high-symmetry edge (armchair or zigzag), allowing the clear separation of the physics of these two common edge types. We find that for all flakes and adatom positions investigated, the Ru magnetic moment prefers to lie in the plane of the island, and that the difference in energy between the most favourable and least favourable in-plane positions for the moment is of the order of 0.1 eV. For adatoms at the edge positions we find that the moment points perpendicular to the edge of the island.

Interestingly, the in-plane versus out-of-plane anisotropy dramatically depends on the edge type, with the zigzag edge showing a marked reduction in both the Ru moment and the corresponding *E*_IO_ as compared to values at the centre of the flake, with the opposite trend seen for the armchair-edge flakes. The origin of this lies in the antiferromagnetic coupling of the adatom to the spin-polarised *p*_z_ edge state in the ZGQD.
